# Rationale for prostaglandin I_2 _in bone marrow oedema – from theory to application

**DOI:** 10.1186/ar2526

**Published:** 2008-10-03

**Authors:** Marcus Jäger, Frank Peter Tillmann, Thomas S Thornhill, Marcus Mahmoudi, Dirk Blondin, Gerd Rüdiger Hetzel, Christoph Zilkens, Rüdiger Krauspe

**Affiliations:** 1 Department of Orthopaedics, Heinrich-Heine University Hospital Duesseldorf, Moorenstrasse 5, D-40225 Duesseldorf, Germany; 2 Clinic for Nephrology and Rheumatology, Heinrich-Heine University Duesseldorf, Moorenstrasse 5, D-40225 Duesseldorf, Germany; 3 Department of Orthopedic Surgery, Brigham and Women's Hospital, Harvard Medical School, 75 Francis Street, Boston, MA 02115, USA; 4 Institute of Diagnostic Radiology, Heinrich-Heine University Duesseldorf, Moorenstrasse 5, D-40225 Duesseldorf, Germany

## Abstract

**Introduction:**

Bone marrow oedema (BME) and avascular osteonecrosis (AVN) are disorders of unclear origin. Although there are numerous operative and non-operative treatments for AVN, pain management in patients with AVN remains challenging. Prostaglandins play an important role in inflammatory responses and cell differentiation. It is thought that prostaglandin I_2 _([PGI_2_] or synonoma prostacyclin) and its analogues promote bone regeneration on a cellular or systemic level. The purpose of this study was to assess the curative and symptomatic efficacy of the prostacyclin analogue iloprost in BME and AVN patients.

**Method:**

We are reporting on 50 patients (117 bones) affected by BME/AVN who were treated with iloprost. Pain levels before, during and 3 and 6 months after iloprost application were evaluated by a visual analogue scale (VAS). The short form(SF)-36 health survey served to judge general health status before and after treatment. Harris Hip Score (HHS) and Knee Society Score (KSS) were performed as functional scores and MRI and X-rays before and 3 and 6 months after iloprost application served as objective parameters for morphological changes of the affected bones.

**Results:**

We found a significant improvement in pain, functional and radiological outcome in BME and early AVN stages after iloprost application, whereas patients with advanced AVN stages did not benefit from iloprost infusions. Mean pain level decreased from 5.26 (day 0) to 1.63 (6 months) and both HHS and KSS increased during follow-up. Moreover, the SF-36 increased from 353.2 (day 0) to 560.5 points (6 months). We found a significant decrease in BME on MRI scans after iloprost application.

**Conclusions:**

In addition to other drugs, iloprost may be an alternative substance which should be considered in the treatment of BME/AVN-associated pain.

## Introduction

Avascular osteonecrosis (AVN) is a common and multifactorial disease, It has a high incidence, estimated to be 15,000 cases of AVN in the femoral head per year in the USA [1]. Frequent risk factors include trauma, steroid therapy or hypercortisonism [2-4], alcohol abuse and different coagulopathies, for example, activated protein C (APC) resistance, protein S deficiency, prothrombin mutations and hyperhomocysteinaemia [5,6]. There are also several rare factors associated with osteonecrosis, such as systemic infection diseases (eg, HIV) [7,8], storage diseases (eg, Gaucher disease) [9], metabolic disorders (eg, hyperuricaemia, hyperlipidaemia) [10,11], sickle cell anaemia [12], aplastic anaemia [13], autoimmune disorders (eg, systemic lupus erythematodes [SLE], rheumatoid arthritis, Behcet's disease) [14], shock and septic syndromes [15], smoking [16], diving [17] and chronic inflammatory bowel diseases. Furthermore, chemotherapy and radiation increase the risk of AVN manifestation in cancer patients [18].

It was shown by Ito and colleagues [19] that there is a correlation between pain and the extent of bone marrow oedema (BME) and that BME is the most significant risk factor for worsening pain. At the time of diagnosis, it is not clear if it is a distinct self-limiting transient condition (ie, BME syndrome, transient osteoporosis) [20-23], a form of reflex sympathetic dystrophy or an early stage of AVN [24]. In addition, subchondral BME is also present in other pathological conditions (eg, tumours, trauma, osteomyelitis) and is also frequently found in osteoarthritis.

Although there is consensus about the different vascular factors that contribute to BME and AVN, the pathogenesis and cause of pain remain unclear. However, the occurrence of associated AVN risk factors, distinct MRI findings, such as a subchondral area of low intensity of at least 4 mm in thickness and 12.5 mm in length, and a prolonged BME for more than 11 weeks correspond to the diagnosis AVN [25].

Advanced stages of AVN can be diagnosed by x-rays showing sclerotic and/or osteolytic areas. Magnetic resonance imaging (MRI) is very sensitive in identifying and characterising BME and AVN in the early stages [26].

The success of different treatment concepts is strongly dependent on the stage of the disease, as classified by the Association Circulation Osseous (ARCO) (Table 1) [27-30]. The treatment options are limited and the long-term prognosis is poor, particularly in advanced bone necrosis. Thus, early diagnosis and rapid, effective treatment are essential. Conservative management consisting of symptomatic therapy has been recommended, especially in cases of BME. It is thought that prostaglandin I_2 _([PGI_2_] or synonoma prostacyclin) and its analogues promote bone regeneration on a cellular or systemic level.

**Table 1:** Classification of avascular osteonecrosis (AVN) as performed by the Association Research Circulation Osseous (ARCO). Diagnostic findings, localisation and extent of AVN are considered. AVN-associated pain usually occurs in late ARCO stages III and IV but can also be found in earlier stages. BME = bone marrow oedema; nps = no pathological signs [27-30]

**ARCO stages**					
	**0**	**I**	**II**	**III**	**IV**
		
**Diagnostic techniques and findings X-ray**	nps	nps	Sclerosis, osteolysis, focal osteoporosis	Crescent sign, flattening of the articular surface (subchondral fracture)	Collapse, joint space narrowing (osteoarthritis)
**CT**	nps	nps	Asterix sign, sclerosis	Subchondral fracture	Collapse, joint space narrowing (osteoarthritis)
**Bone scan**	nps	Cold spot	Cold in hot spot	Hot in hot spot	Hot spot
**MRI**	nps	BME	Osteonecrosis, reactive interface	Subchondral fracture	Collapse, joint space narrowing (osteoarthritis)
		
**Subclassification**	No	- medial			No
		- central			
		- lateral			
		
**Quantification**	No	% area involvement:	Length of crescent:	% of surface collapse and dome depression	No
		Minimal A: <15%	A: <15%	(A, B, C)	
		Moderate B: 15 to 30%	B: 15 to 30%		
		Extensive C: >30%	C: >30%		

Preliminary promising results in the literature [31-37] and in our own experience [38,39] encouraged us to conduct a prospective study to investigate the curative potential and analgetic efficiency of the vasoactive prostacyclin analogue iloprost. The stable prostacyclin analogue iloprost is approved for treatment of critical ischaemia occurring secondarily to peripheral arteriosclerotic obliterative disease of diabetic angiopathy (intermittent claudication). Furthermore, iloprost is administered as an inhalative for patients with pulmonary arterial hypertension [40]and the application of iloprost in systemic sclerosis is currently under investigation in clinical trials [41]. Other rare indications for iloprost are severe bone pain caused by sickle cell crisis [36], Raynaud's phenomena [42] and SLE [42,43]. Moreover, it has been shown that iloprost improved preservation in organ storage in transplantation surgery for heart, liver, lungs and kidneys [44,45].

## Materials and methods

### Patients

Between October 2002 and December 2005, 61 patients with painful BME or AVN (mean (SD) age = 45.9 (14.9) years; range = 11 to 76 years) were treated with iloprost. According to the study protocol, we carried out a prospective, MRI-controlled observational study on 50 patients (mean age = 45.2 (14.2) years; range = 24 to 76 years; sex ratio: 22 men to 28 women) with symptomatic AVN or painful BME. The average body weight was 73.5 (14.1) kg and the mean height was 172.0 (9.4) cm. All AVN were associated with an almost distinctive BME, which showed a high variability in extent and was not evaluated separately.

Patients aged between 18 and 80 years with painful BME and additional AVN risk factors or BME persisting for more than six months or AVN stage greater than ARCO I were included in the study. Patients were excluded if they had acute or chronic infections or hypertension with systolic values higher than 160 mmHg, or those who had ischaemic heart attacks or cerebral ischaemia/bleeding within the past six months or surgery within the past six months or bleeding disorders, or if the women were pregnant or breastfeeding. Based on MRI scans, x-rays and clinical examinations, patients with osteoarthritis, joint instabilities and axis deformities 10° more than the statistical normal were also excluded. The study protocol was approved by the local Ethics Committee (local ethical committee of the Heinrich-Heine-University, Düsseldorf, trial number: 2355) and included written informed consent according to the Declaration of Helsinki in its present version.

### Parameters

Iloprost (Ilomedin; Schering AG, Germany) was dissolved in 0.9% saline solution and applied intravenously over a period of six hours per day in a weight-related schedule for a total of five days (Table 2).

**Table 2:** Detailed iloprost infusion scheme. The body weight-dependent dose was increased from day one to day five. At day five and four, the dose was adjusted according to adverse effects. The infusion time was six hours per day

	First day (mL/hour)	Second day (mL/hour)	Third to fifth day (mL/hour)
Body weight (kg)	(0.5 ng/kg/minute)	(0.75 ng/kg/minute)	(1.0 ng/kg/minute)

60	2.2	3.4	4.5

70	2.6	4.0	5.3

80	3.0	4.5	6.0

90	3.4	5.1	6.8

100	3.8	5.7	7.5

110	4.1	6.2	8.3

Based on medical history and clinical examination, the Harris Hip Score (HHS), the Knee Society Score (KSS) and assessment of pain level on a visual analogue scale (VAS) served for evaluation during a follow-up of up to six months. The VAS is classified from 0 (no pain) to 10 (severe pain). Moreover the short form (SF)-36 health survey was used to assess patients' health status. It is the short form of an instrument developed for the Medical Outcome Study and contains 36 items that can be aggregated to eight scales [46].

In addition to clinical parameters, plain radiographies in two standard planes (one when weight bearing) and MRI scans (T1 weighted, T2 weighted and short T1 inversion recovery (STIR) weighted) were performed for radiographic analysis by a blinded independent radiologist (DB) (parameters: ARCO stages, extent of BME: progression, persistence, regression). Table 2 shows the infusion scheme and table 3 gives an overview of the study design (Table 3).

**Table 3:** Study design to evaluate the therapeutic potential of iloprost over a follow-up of six months. Clinical parameters and MRI evaluation of the patients before and three and six months after iloprost application. X: investigation; -: no investigation

***Follow-up***	***Before treatment (day 0)***	***Day one to five***	***Three months***	***Six months***
Clinical parameters				
Visual analogue pain scale	**X**	**X**	**X**	**X**
Harris-Hip-Score	**X**	-	**X**	**X**
Knee-Society Score	**X**	-	**X**	**X**
SF-36	**X**	-	**X**	**X**

Radiological parameters				
X-rays in two planes	**X**	-	**X**	**X**
MRI scans	**X**	-	**X**	**X**

Any side effects and adverse events were recorded. Uneventful effects during or after iloprost therapy were recorded and classified as severe (hypotension, arrhythmia, bleeding, thromboembolism, myocardial insufficiency, acute respiratory distress syndrome, pulmonary oedema, allergic reactions with systemic clinical signs, shock) and minor (flush, erythema, headaches, nausea) side effects.

### Statistical analysis

Student's *t*-test for independent statistical groups was used for statistical analysis: p < 0.01 was highly statistically significant, p < 0.05 was statistically significant and p > 0.05 showed no significance. The average values, standard deviations and the range from minimum to maximum readings served as descriptive parameters at follow-up examinations. Connections between the different parameters were recorded and determined by linear regression analysis.

## Results

**Figure 1:**
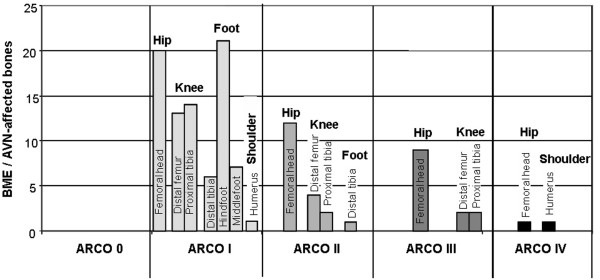
**Distribution of 117 bone marrow oedema (BME)/avascular osteonecrosis (AVN)-affected bones (98 joints) and Association Research Circulation Osseous (ARCO) stages according to roentgenological and MRI-based diagnosis**. Before treatment with iloprost the hip joint was affected in 43%, followed by foot joints in 28%, the knee joint in 26% and the shoulder in 3%. The initial ARCO distribution was as follows: No ARCO 0, 82 ARCO I, 20 ARCO II, 13 ARCO III and two ARCO IV.

Overall, 117 bones (98 joints) in patients in this study were affected by BME or AVN before treatment. Figure 1 shows the regional distribution and ARCO stages of the 50 patients in this study. Considering medical history, we found different associated risk factors for BME and AVN: nicotine abuse (10 patients), steroid medication (25 patients), trauma (four patients), hyperlipoproteinaemia (three patients), activated protein C resistance (one patient) and chemotherapy (one patient). No risk factors were found in 26 patients (idiopathic AVNand BME). We found different AVN stages on MRI and x-ray evaluations in two standard planes. Classified by ARCO, there were 82 ARCO I bones, 20 ARCO II bones, 13 ARCO III bones and two ARCO IV bones.

No severe adverse effects were observed in any patients during intravenous iloprost administration. In two patients, severe headaches occurred on infusion day four and led to early termination of iloprost therapy. We observed one thrombophlebitis at the injection site, which was treated with antiseptic patches and healed within four days. Flushes or erythemas occurred from day three in 90% of patients during infusion.

**Figure 2:**
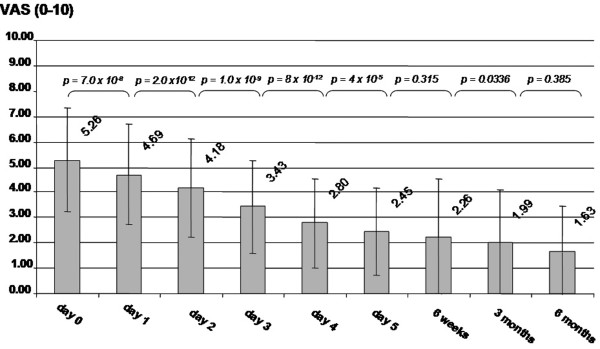
**Follow-up of 50 patients in pain level measured by visual analogue scale (VAS) from 0 (no pain) to 10 (severe pain)**. The graph shows progressive improvement in pain for patients with bone marrow oedema/avascular osteonecrosis during and after iloprost application.

Iloprost showed a highly significant reduction in the level of pain evaluated by VAS during intravenous application within five days starting from 5.3 (sd = 2.0; range = 2 to 10) before treatment (day 0) to 2.5 (sd = 1.7; range = 0 to 6) on average at day five. There was still an improvement in pain three and six months after infusion corresponding to a pain level on the VAS (At three months = 2.0; sd = 2.1; range = 0 to 8: At six months = 1.6; sd = 1.8; range = 0 to 7) but the reduction in pain in this period was not statistically significant (p > 0.05). Starting at day three, about 60% of all patients reported intermediate "gnawing and dull" sensations in the affected bones during iloprost application. These dysesthesias disappeared spontaneously within six hours when infusion was stopped. Figure 2 shows the outcome in pain over a six months of follow up.

**Figure 3:**
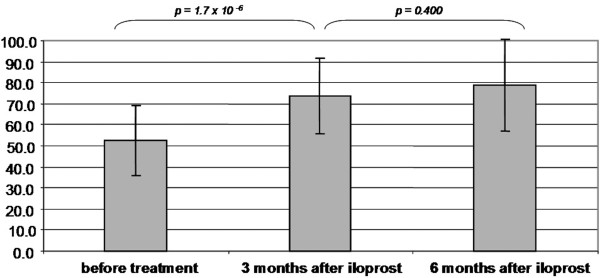
The graph shows the average values in Harris-Hip-Score of bone marrow oedema/avascular osteonecrosis patients before and at three and six months after treatment with iloprost.

**Figure 4:**
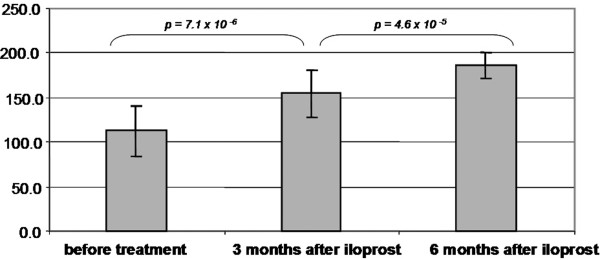
The graph shows the average values in Knee-Society-Score of bone marrow oedema/avascular osteonecrosis patients before and at three and six months after treatment with iloprost.

There was a highly significant improvement in the mean HHS from 52.6 points (sd = 16.5 points; range = 23 to 84 points) before treatment to 73.6 points (sd = 17.9 points; range = 39 to 99 points) after three months and 79.9 points (sd = 21.9; range = 26 to 100 points) after six months. In the period between three and six months after iloprost infusion, the HHS improvement was not significant (p > 0.05) as shown in figure 3. Furthermore, the KSS increased from 112.8 points (sd = 28.5 points; range = 60 to169 points) to 154.7 points (sd = 26.2; range = 100 to 190 points) at three months and to 186.4 points (SD = 14.3; range = 158 to 200 points) at six months (figure 4).

**Figure 5:**
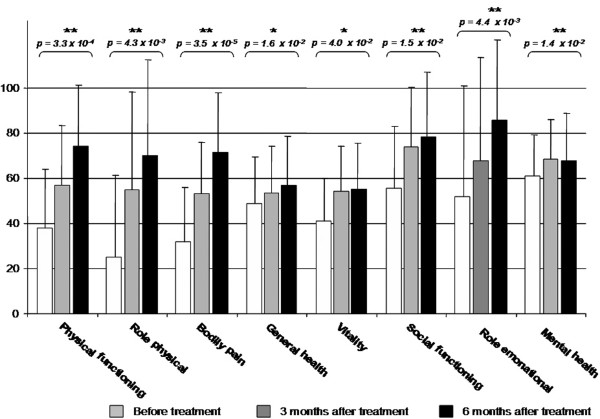
**The SF-36 health survey showing improvement in all eight scales**. There was a highly significant improvement in physical functioning, role physical, bodily pain, social functioning, role emotional and mental health after iloprost application in bone marrow oedema/avascular osteonecrosis patients. General health and vitality show a significant improvement at the six-month follow-up. *: significant (p < 0.05); **: highly significant (p < 0.01).

Corresponding to a better functional outcome and a significantly lower pain level in BME and AVN patients, quality of life evaluated by SF-36 score showed significant improvement during and after iloprost infusion (figure 5). The average (sd) values for SF-36 were 353.2 (12.3) points before treatment, 483.7 (8.3) points three months after infusion and 560.5 (10.2) points six months after iloprost application. A highly significant improvement was seen in physical functioning, role physical, bodily pain, social functioning, role emotional and mental health before and after six months of iloprost infusion, and the general health and vitality scales showed a significant improvement. However, after three months we found no significant improvement in vitality and general health, and there was a reduction in mental health scores from month three to six with no significance.

**Figure 6:**
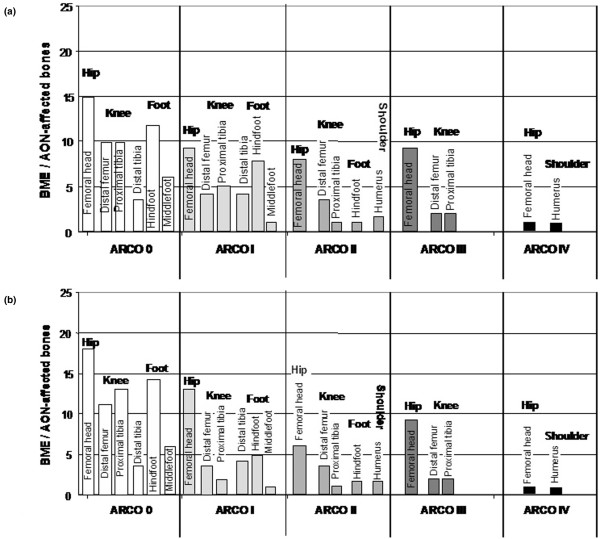
**The graph shows the different osteonecrosis stages according to Association Research Circulation Osseous (ARCO) classification during follow-up**. (a) ARCO stages three months after iloprost application. The distribution was as follows: 56 ARCO 0, 31 ARCO I, 15 ARCO II, 13 ARCO III and two ARCO IV. (b) ARCO stages six months after iloprost application. The distribution was as follows: 65 ARCO 0, 23 ARCO I, 14 ARCO II, 13 ARCO III and two ARCO IV.

**Figure 7:**
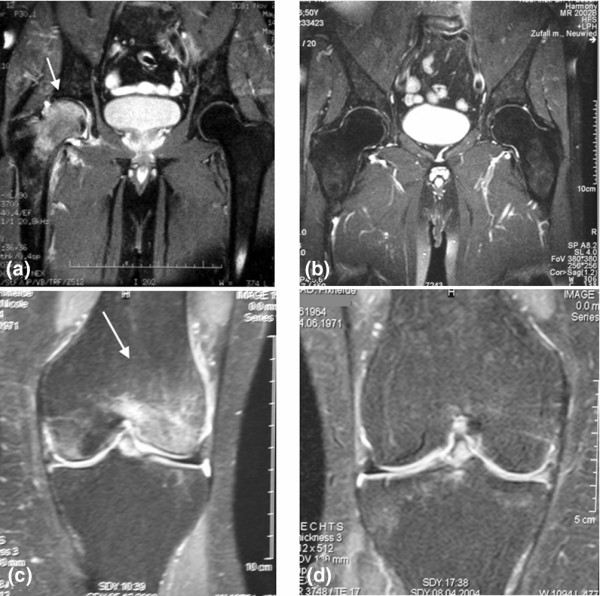
**MRI scans (T2-weighted) of two different patients with bone marrow oedema (BME) (a, c) before and (b, d) six months after iloprost application**. (a, b) The BME of a 50-year-old man with chronic alcohol abuse resolved completely after iloprost infusion. (c, d) A 32-year-old woman with painful BME of the medial condylus during immunosuppressive therapy after kidney transplantation was treated with iloprost and healed within six months.

The clinical findings during follow-up correspond to the MRI findings. After three and six months, MRI scans showed a significant decrease in the extent of BME. Overall, 65 of 117 affected bones were free of BME within six months of iloprost application. In contrast to a significant decrease in BME and early AVN stages, advanced AVN stages (ARCO III and IV) were not influenced by iloprost; however, in some patients with ARCO stages III and IV iloprost showed an analgetic effect. Figures 6a and 6b and table 4 show detailed data of MRI follow up after iloprost application and figure 7 shows MRI findings of two typical patients with BME before and after iloprost infusion.

**Table 4:** Follow-up of 117 bones affected by bone marrow oedema/avascular osteonecrosis before and three and six months after intravenous iloprost application. There is a significant improvement in Association Research Circulation Osseous (ARCO) I and early ARCO II stages

	**ARCO 0**	**ARCO I**	**ARCO II**	**ARCO III**	**ARCO IV**
	**Femoral head (n = 42)**				

Before treatment	0	20	12	9	1
Three months	15	9	8	9	1
Six months	18	8	6	9	1
					
	**Distal femur (n = 19)**				

Before treatment	0	13	4	2	0
Three months	10	4	3	2	0
Six months	11	3	3	2	0
					
	**Proximal tibia (n = 18)**				

Before treatment	0	14	2	2	0
Three months	10	5	1	2	0
Six months	13	2	1	2	0
					
	**Distal tibia (n = 7)**				

Before treatment	0	6	1	0	0
Three months	3	4	0	0	0
Six months	3	4	0	0	0
					
	**Hindfoot (n = 21)**				

Before treatment	0	21	0	0	0
Three months	12	8	1	0	0
Six months	14	5	2	0	0
					
	**Middlefoot (n = 7)**				

Before treatment	0	7	0	0	0
Three months	6	1	0	0	0
Six months	6	1	0	0	0
					
	**Humerus (n = 3)**				

Before treatment	0	1	1	0	1
Three months	0	0	2	0	1
Six months	0	0	2	0	1

The regression analysis reflects the strong negative correlation between pain level reduction and functional outcome, life quality and reduction of BME in MRI scans. The correlation coefficient between pain and the HHS was -0.99, between pain and the KSS it was -0.96, and between pain and the SF-36 it was -0.91. There were no substantial iloprost-mediated effects on joint cartilage in standard MRI sequences.

## Discussion

During intravenous treatment with the prostacyclin analogue iloprost, a highly significant reduction of pain in patients with BME and/or AVN could be demonstrated. Moreover, iloprost showed a non-significant but progressive reduction of pain through to the last follow-up examination. As shown in the results, the anti-oedema effects of iloprost were dependent on the ARCO stage of the AVN. Patients in early ARCO stages I and II especially benefited from iloprost application with respect to pain relief, functional outcome and BME reduction. Although iloprost has a short half-life *in vivo *of about 25 minutes, the clinical and MRI findings were not only short-term effects but lasted until the final follow up at six months after application. The high number of patients with multifocal AVN (50 patients, 117 bones) in our study is partly due to the fact that 20% of individuals underwent kidney transplantation. All of these patients developed a multifocal painful BME ('posttransplant distal limb syndrome') [47].

Our results correspond to the data from other investigators. Disch and colleagues [35] reported on 16 patients with BME and 17 patients with AVN of the proximal femur who were treated with iloprost. They demonstrated a significant improvement in functional outcome measured by HHS (p < 0.001), a reduction in extent of BME and pain relief over 12 weeks. In another study, Aigner and colleagues [32] investigated the effects of intravenously applied iloprost on 38 hips with BME in the femoral head and compared these results with core decompression. The iloprost group achieved better results after a mean follow-up of 11 months. After iloprost application, pain at rest was no longer present within a mean of eight days and pain during exercise took four weeks to normalise. Meizer and colleagues [37] reviewed 104 patients with painful BME after intravenous iloprost therapy over four months in an MRI-controlled study. At follow-up, pain reduction was detected in 64% of all patients and 65% of the subjects had a significant reduction in BME size or complete normalisation. Also other recent study supported the effectiveness of prostaglandin (PG) I_2 _analogue iloprost in BME and/or AVN [39,48,49].

As an alternative treatment concept, some authors report good results after core decompression, based on the theory that AVN-associated pain is due to elevated intramedullary pressure [16,50,51]. Although core decompression can lead to rapid and complete relief from symptoms and resolution of the changes seen on MRI, some authors underline the perioperative risks including fractures, damage to cartilage, persisting haematomas and local infections. In addition, six weeks of partial or no-weight-bearing and physiotherapy are usually required after core decompression. Based on histological studies, there is a high failure rate to achieve the correct position of the drill channels after femoral head core decompression [20,23,52]. It is not possible to control and define the destination of the drill wires in early stages of AVN and BME using fluoroscopy, so the risk of dislocation is especially high in these stages. Considering the data from Wang and colleagues [53] extracorporeal shock-wave therapy may be another therapeutic option in the treatment of AVN-associated pain, but it can also induce AVN as reported by Durst and collagues [54]. In particular, the high energy extracorporeal shock-wave application on bones is associated with pain caused by microtrauma or microfracture and haematoma and requires sufficient anaesthesia during treatment [55].

The pharmacokinetic effects of iloprost that lead to better perfusion in tissue with a critical blood supply are multiple. It induces vasodilation and has an impact on rheological properties of the terminal vascular bed [56]. Moreover, it reduces capillary permeability, inhibits platelet aggregation and diminishes the concentration of free oxygen radicals and leukotrienes [57-60]. However, the pharmacological effects that are responsible for the relief of pain and a decrease in BME are not yet known and remain controversial. It is unclear if the pain relief and reduction in extent of BME during and after iloprost application are primarily based on a normalisation of intraosseous pressure or on interactions with local leukotrienes and cytokines.

**Figure 8:**
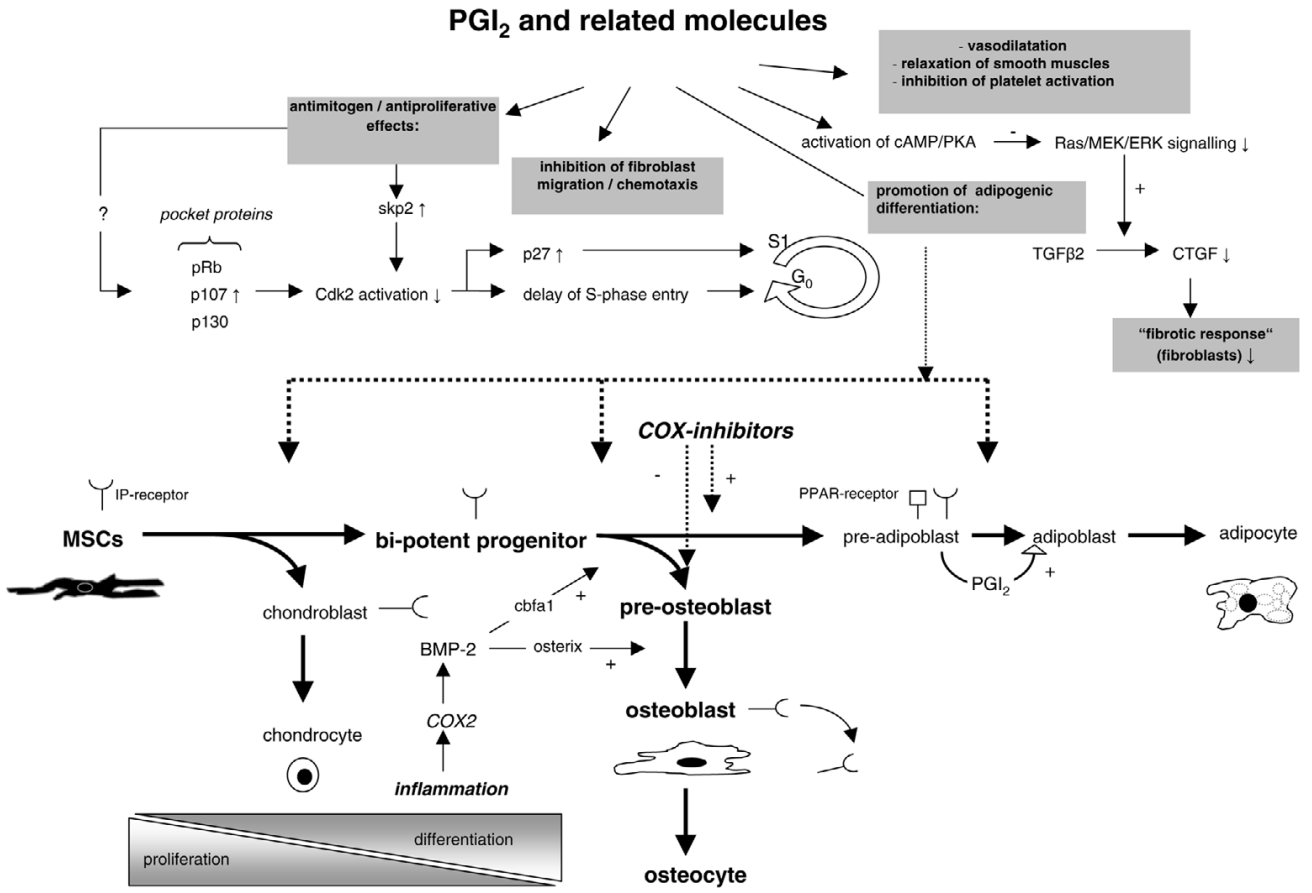
The figure gives an overview of prostacyclin (PGI_2_) and related molecules in mesenchymal cell proliferation and differentiation.

**Figure 9:**
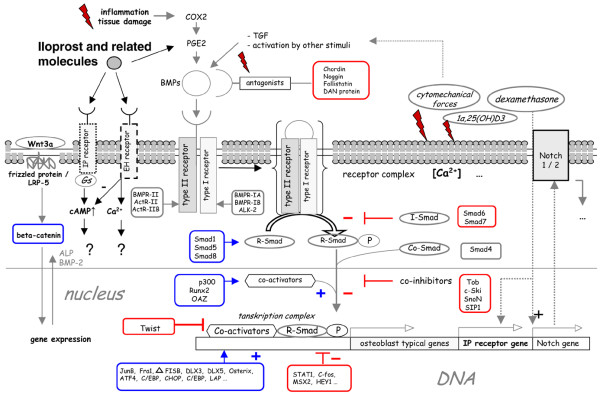
**The scheme summarises different intracellular pathways during osteoblast differentiation which include promoting (blue) and inhibiting (red) factors and are mainly mediated by Smad proteins**. Based on recent studies it can be assumed that not only the TGFβ/BMP, the Wnt/beta-catenin, the Notch pathway or direct gene activations by steroids but also prostaglandins are involved in osteoblast differentiation. Prostacyclin and its related molecules, such as iloprost, play a crucial role in various cellular mechanisms and control intracellular signals: the IP receptor mediates the actions of its ligand via Gs (or G_q_, G_11_, G_i_) protein activation leading to adenylate cyclase activation followed by an intracellular increase of cAMP. Moreover, the human IP receptor gene presents various bindings sites such as glucocorticoid response element (GRE) and it can be concluded that glucocorticoids also regulate its expression. In addition, the human EP receptors with its various subtypes such as EP_1–4 _and their isoforms are also activated by iloprost. One major EP-related effect is the mobilisation of intracellular Ca^2+ ^but also a down-regulation of intracellular cAMP concentration as described for the EP_4 _receptor. However, the complex intracellular effects that are triggered by iloprost and the importance in osteoblast differentiation are poorly understood to date.

From a molecular point of view, the G-protein-coupled prostanoid IP receptor plays a crucial role in the prostacyclin-induced effects. Activation of IP receptors may result in pain sensation, inflammatory responses, inhibition of platelet aggregation and vasodilation in vascular tissue [61]. Furthermore, it has been shown that prostacyclin (PGI_2_) is an important mediator implicated in bone metabolism which acts via the kinase A-pathway as a potent inhibitor of bone resorption and mediates bone modelling [62]. Although the specific effects of PGI_2 _on its IP receptor are well documented, there are few data available in the literature about the distribution of IP receptors in human bone. Fortier and colleagues [62] detected IP receptors in fetal and adult osteoclasts and osteoblasts. In contrast to fetal osteocytes, adult osteocytes do not express the IP receptor. Moreover adult osteoblasts lose the IP receptor when these cells are trapped in the bone matrix. As demonstrated by Fortier and colleagues [62], IP receptors show a perinuclear distribution in osteoblasts, but are not frequently seen in multinuclear osteoclasts. Furthermore, there is no difference in the expression of IP receptors in pagetic, osteoporotic and normal bone. Aubert and colleagues [63] demonstrated that IP receptors play a crucial role in preadiposing cell stimulation and differentiation. Figures 8 and 9 give a schematic overview of some PGI_2_-mediated effects.

The IP receptor plays an important role in rat dorsal root ganglion (DRG) neuron sensitisation, which is measured by the release of the neurotransmitter substance P. Nakae and colleagues [64] showed that the IP antagonist 2-[4-(1H-indol-4-yloxymethyl)-benzyloxycarbonylamino]-3-phenyl-propionic acid (compound A) inhibits the accumulation of the second messenger cAMP in the rat osteosarcoma cell line and primary cultured rat DRG neurons without affecting other eicosanoid receptors and leads to an iloprost-induced reduction in the release of substance P.

The interpretation of an osteoblast-protective effect caused by the prostacyclin analogue iloprost and its clinical relevance for pain relief in AVN is critical because the molecular pathways are complex. Other agents, such as the stable analogue carbacyclin (cPGI_2_), BMY 45778 and cicaprost, are also potent agents with IP-receptor binding properties and may influence pain [63,64].

The results of this study and our experience with more than 60 BME patients showed that pain associated with BME and AVN can sufficiently be reduced by iloprost application. Our findings confirm those of other investigators that iloprost has a curative potential in ARCO I and early II AVN stages in adults. Although children with early stages of AVN have been successfully treated with iloprost in a pilot study [65], it is unclear if this vasoactive drug would be appropriate in healing juvenile AVN (eg, Perthes' disease).

Cartilage damage classically occurs late in AVN in response to mechanical strain caused by fracture of the subchondral sequestrum. Therefore, the joint space in conventional x-rays remains normal for longer in AVN patients compared with those with osteoarthritis. To prevent progression of AVN that may result in an early total joint replacement, we recommend an early MRI diagnosis to detect BME or early-stage AVN in cases of joint pain of unknown origin. In MRI, BME appears as high-signal intensity on T2-weighted images, best seen if fat suppressed, and as low-signal intensity on T1-weighted images. In early studies, these findings were described as a diffuse or homogeneous pattern of AVN, but lesions are often uncharacteristic, particularly in an early stage [27,29]. An interface between areas of osseous resorption and healing is often present on MRI and is referred to as the double-line sign. The sign is created by a high-signal intensity line, representing hyperaemic tissue, immediately apposed to a low-signal intensity line, representing sclerotic bone. This characteristic MRI sign is seen in irreversible early-phase AVN, representing stage II of the ARCO classification (Table 1). MRI has been found to be the most sensitive method of detecting the presence of early AVN [26,66,67].

However, there is controversy about whether BME represents a distinct self-limiting disease (also known as transient osteoporosis), transient marrow oedema or merely reflects a subtype of AVN. The diagnosis of BME syndrome can only be made retrospectively, regardless of whether a progressive AVN or a BME syndrome will follow subsequently. Considering the similar clinical presentation of BME and AVN and the high variance in prognosis and therapeutic consequences between self-limiting BME syndrome and progressive AVN, we recommend applying iloprost with preference in BME patients with additional AVN risk factors or in persisting and painful BME.

We showed that osteonecrosis-associated pain can be treated sufficiently by the prostacyclin-analogue iloprost. Therapy in bone BME and AVN was only promising in early AVN stages (ARCO I and II). In addition to other drugs, iloprost may be an alternative substance that should be considered in the treatment of BME and osteonecrosis-associated pain. Further controlled clinical studies are needed to show if iloprost has any effects on joint cartilage and to investigate if osteoarthritis-related BME is an indication for future application of PGI_2 _and related molecules. Appropriate MRI sequences such as dGEMRIC, which not only allow evaluation of bone-related effects but may also help to detect molecular changes within the complex cartilage histo-architecture, should be used in further investigations.

## Conclusion

Iloprost improves the functional and radiological outcome in patients with BME. However, advanced AVN stages (≥ ARCO II) are irreversible and iloprost is not potent enough to heal the osteonecrotic areas. Although the individual risk for severe side effects during iloprost treatment is low when the dosage is adapted to the body weight, contraindications should be considered carefully.

## Abbreviations

ARCO: Association Research Circulation Osseous; AVN: avascular osteonecrosis; BME: bone marrow oedema; DRG: dorsal root ganglion; HHS: Harris Hip Score; KSS: Knee Society Score; MRI: magnetic resonance imaging; PG: prostaglandin; SF: short form; STIR: short T1 inversion recovery; SLE: systemic lupus erythematodes; VAS: visual analogue scale.

## Competing interests

The authors declare that they have no competing interests.

## Authors' contributions

MJ worked out the design of the study and carried out the clinical investigations of the probands. FPT and GRH drafted the manuscript and performed part of the clinical investigation. TST edited the manuscript. MM, CZ and RK participated in the coordination and clinical investigation of the patients. DB carried out the evaluation of the x-rays and MRI scans. All authors read and approved the final manuscript.
